# Partitioning the effects of regional, spatial, and local variables on beta diversity of salt marsh arthropods in Chile

**DOI:** 10.1002/ece3.4922

**Published:** 2019-01-30

**Authors:** Cristina Coccia, José Miguel Fariña

**Affiliations:** ^1^ Center of Applied Ecology and Sustainability (CAPES) Pontificia Universidad Católica de Chile Santiago Chile; ^2^ Department of Ecology and Evolutionary Biology Brown University Providence Rhode Island

**Keywords:** arthropod, beta diversity, Chile, nestedness, salt marsh, turnover

## Abstract

**Aim:**

We examined the influence of regional, spatial, and local variables (edaphic characteristics and vegetation structure) on patterns of arthropod variation along the Chilean coast by partitioning beta diversity into its turnover and nestedness components.

**Location:**

2,000 km along the coast of Chile.

**Methods:**

We collected ground‐dwelling arthropod samples from nine marshes during two seasons. A clustering method was used to examine patterns of arthropod similarity across salt marshes. We also calculated multiple‐site beta diversity and partitioned it into its turnover and nestedness components. Variation partitioning was then used to identify the major drivers of their variation (regional, spatial, and local variables). We compared results for the whole arthropod community and for the most abundant, speciose, and functionally different groups, Crustacea, Coleoptera, and Araneae.

**Results:**

Salt marsh arthropod similarities did not depend on the geographic proximity of sites. Arthropod beta diversity was mainly determined by its turnover component. A significant fraction of community variation was related to the spatially structured variation of climate or edaphic factors. However, the exclusive contribution of spatial variables had also a role.

**Main conclusions:**

Each salt marsh on the Chilean coast has the capacity to accommodate unique invertebrate taxa. Species sorting along the climatic gradient together with dispersal‐based processes seems the key structuring force of the arthropods and Crustacean variation in the marshes we studied, while species sorting alone might be more important for Coleoptera variation.

## 1 INTRODUCTION

Beta diversity, the variation of species composition among sites (Koleff, Gaston, & Lennon, 2003), is a key factor to understand broad‐scale patterns of biodiversity and for conservation planning (Kraft et al., 2011; Qian, Ricklefs, & White, 2005; Socolar, Gilroy, Kunin, & Edwards, 2016). Most studies on the topic have focused on understanding the effect of regional (i.e., climate) and geographic factors (e.g., spatial distances) on the assemblage composition of several taxa including plants, insects, and vertebrates (Baselga & Valverde, 2007; Qian & Ricklefs, 2007; Rodrigues & Diniz‐Filho, 2017; Svenning, Fløjgaard, & Baselga, 2011), and more recently also historical factors (Dobrovolski, Melo, Cassemiro, & Diniz‐Filho, 2012; Murphy et al., 2015). By comparison, biotic factors (e.g., species interactions), which are well‐known drivers of species assemblages at the local scale, have received little attention in large‐scale studies, although they clearly may set species range limits (Schemske, Mittelbach, Cornell, Sobel, & Roy, 2009; Wisz et al., 2013).

Among the different types of species interaction (e.g., competition or consumption), habitat structures and ecosystem functions provided by living organisms constitute a biotic influence of foundation species (sensu Dayton, 1972; Ellison et al., 2005) on the diversity of others, and as a factor, it may have an influence at both local (i.e., species interactions) and regional/geographic scales (their relation to climate and other abiotic factors). For example, the structural complexity of vegetation is known to affect the distribution and interaction of the associated biota either directly, by providing food and shelter against predators, or indirectly, by altering edaphic factors (Denno, Finke, & Langellotto, 2005), and in turn itself is partly related to geographic variation in climatic conditions. As a consequence, vegetation structure can be expected to mediate species distribution and should be taken into account in biogeographic studies.

Differences in community composition across space and time are the result of two different but not mutually exclusive processes: species replacement from one site to another (turnover) and species lost from one site to another (nestedness) (Baselga, 2010; Harrison, Ross, & Lawton, 1992). Only recently have studies begun to assess the relative importance of turnover and nestedness over broad spatial scales (Dobrovolski et al., 2012; Leprieur et al., 2011; Svenning et al., 2011; Viana et al., 2016), owing to significant methodological advances (Baselga, 2010). The relative contribution of these two components has been shown to be useful for understanding what causes beta diversity patterns at large scales (Dobrovolski et al., 2012; Leprieur et al., 2011; Svenning et al., 2011). So far, turnover has been frequently found to be the most important driver of beta diversity in both aquatic and terrestrial ecosystems at large scales (Schmidt et al., 2017; Viana et al., 2016). However, processes responsible for shaping species replacement are still poorly explored (Baselga, 2010; Schmidt et al., 2017; Soininen, Heino, & Wang, 2018). Partitioning beta diversity into its two components and relating them to the factors potentially responsible for their variation can thus provide deeper insights into the mechanisms responsible for community organization.

Coastal salt marshes are excellent model systems for studying large‐scale variation in species composition, especially for plants because of the simplicity of their community (Bertness & Ewanchuk, 2002; Fariña, He, Silliman, & Bertness, 2017; Pennings, Siska, & Bertness, 2001). Salt marshes along the Chilean coast exhibit strong climatic gradients. As latitude decreases, the environment becomes more stressful because precipitation decreases and temperature increases. As a result of these gradients, salt marshes on the coastline of Chile have different soil salinities and show a transition in the dominant vegetation from *Sarcocornia fruticosa* at higher latitudes to *Spartina densiflora *at low latitudes (Fariña et al., 2017). These are the most common salt marsh halophytic plants (Isacch et al., 2006). However, the two species have different growth forms: *S. fruticosa* is a semi‐woody dicot with procumbent to erect succulent stems (Pellegrini, Konnerup, Winkel, Casolo, & Pedersen, 2017; Scarton, Day, & Rismondo, 2002), while the saltgrass *S. densiflora* has a bunchgrass‐like growth form with erect and overlapping branches (DiTomaso et al., 2013).

Arthropods are one of the major components of salt marsh biodiversity and participate in key ecosystem processes such as nutrient cycling and impact primary production (Pennings, McCall, & Hooper‐Bui, 2014); they may constitute a link between terrestrial and aquatic food webs. Arthropods are sensitive to changes in the physical (e.g., temperature and precipitation) and chemical (e.g., soil salinity) structure of their environments (Desender & Maelfait, 1999; Irmler, Heller, Meyer, & Reinke, 2002; Pétillon et al., 2008; Southwood, Brown, & Reader, 1979), and some of them (e.g., spiders; ground‐dwelling beetles) require specific vegetation structure for feeding and refuge (Brose, 2003; Pétillon et al., 2008). Arthropods have a wide variety of feeding strategies, from parasites to predators, and their dispersal abilities vary across taxa. These characteristics make them ideal to investigate which factors are more important in structuring patterns of their beta diversity at geographic large scales. Given the threats suffered by coastal wetlands worldwide (Davidson, 2014) and considering the key role of arthropod fauna on their functioning, understanding the factors affecting arthropod distribution is an important first step toward their protection, those of the wetland functions and of the services they provide.

Nonetheless, in Chile, as in other countries arthropod studies have usually been surveys of the fauna at a particular site (Meserve, Vásquez, Kelt, Gutiérrez, & Milstead, 2015; Pizarro‐Araya et al., 2012), and there is no previous information on the factors influencing their biogeography, especially in salt marshes where the available information about arthropods is pitifully small.

Our main objectives in this study were to investigate the geographic variation of salt marsh arthropod structure and composition along 2,000 km of the Chilean coast and to examine the influence of regional, spatial, and local variables (edaphic characteristics and vegetation structure) on patterns of nestedness, turnover, and overall beta diversity of the arthropod community. To test these hypotheses, we compared geographic patterns of the overall arthropod community with those of the most speciose and/or abundant taxa (Crustacea, Coleoptera, and Araneae), which are ecologically and functionally different. Terrestrial Crustacea are detritivores and poor dispersers, ground‐dwelling Coleoptera include taxa with a wide variety of feeding habits and dispersal abilities, and Araneae include generalist predators that are mostly passive aerial dispersers. Neighboring marshes should be more similar than geographically distant marshes because of the similar climate and environmental conditions. Based on other broad‐scale studies in both terrestrial and aquatic systems (Dobrovolski et al., 2012; Viana et al., 2016), we expect that spatial turnover will contribute more to the overall beta diversity than nestedness in each group. Lastly, we expect arthropod beta diversity to be explained mainly by local vegetation structure.

## 2 MATERIALS AND METHODS

### 2.1 Study sites

**Figure 1:**
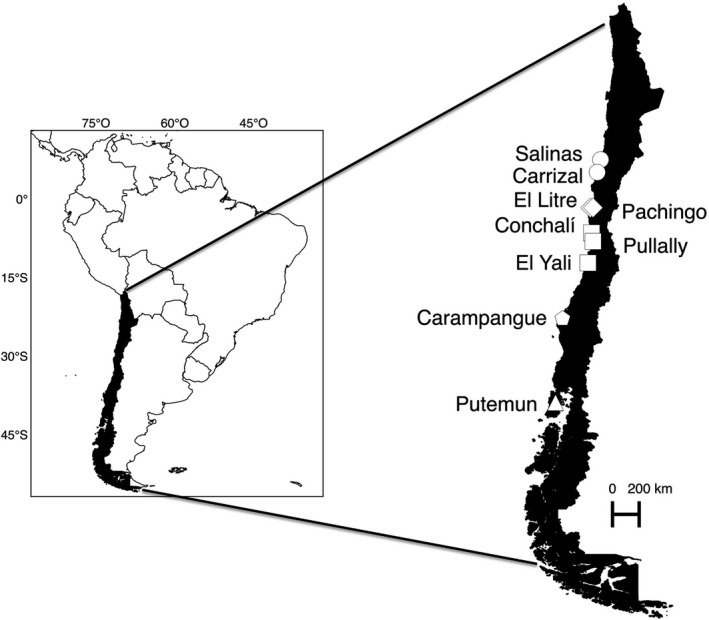
Map of the studied marshes along the Chilean coast. Circles = hyperarid region; diamond = arid region; squares = semi‐arid region; pentagon = humid region; triangle = hyper‐humid region

We studied nine marshes along 2,000 km of the Chilean Pacific coast from Copiapó (27°S) to Chiloé (42°S) (Figure 1). These marshes spanned over five climate regions of Chile, the hyperarid (2), arid (2), semiarid (3), humid (1), and hyper‐humid (1) regions from north to south, respectively (Santibañez & Santibañez, 2008). These marshes show a gradual decrease in mean annual temperature but increase in mean annual precipitation with latitude (Fariña et al., 2017 and Supporting Information Appendix S1). All the selected marshes were located at the mouth of rivers but had variable connections to the sea and were exposed to semidiurnal tides but with different ranges and frequencies (see Fariña et al., 2017 for details). The highest values were recorded in the southernmost salt marshes of Putemun (Fariña et al., 2017).

Sampling was conducted during autumn and spring 2016 (twice in total) within the high marsh elevation area, which is the zone above the highest level of tidal inundation.

### 2.2 Invertebrate sampling and processing

During each visit, ground‐dwelling invertebrates were collected using 12 pitfall traps distributed randomly within the high marsh zone at ca. 5 m from each other to avoid mutual influence. Each trap consisted of two plastic cups (each 9 cm diameter and 12.5 cm deep) inserted one inside the other in the ground and partially filled with a 50% ethylene glycol and 50% water solution as preservative. The traps were emptied after 72 hr.

In the laboratory, samples were washed, sieved (250 μm mesh), and preserved in 70% ethanol. Invertebrates were identified under a stereomicroscope. The most numerous and/or speciose taxa belonging to Crustacea, Coleoptera, and Araneae were identified to the lowest practical taxonomic level using the available keys (Peña, 1971; Roig‐Juñent & Domínguez, 2001; Vidal & Guerrero, 2007) and with expert taxonomic assistance (see Acknowledgments). The less numerous Hemiptera, Hymenoptera, Lepidoptera, Diptera, Myriapoda, Orthoptera, Thysanura, and Gastropoda were identified to family level. Aerial taxa (e.g., adult Diptera, Apidae, and Lepidoptera) were not included in data analyses as pitfall traps are not an appropriate sampling method for these taxa. Immature spiders, larvae, and nymphs were excluded from analyses because accurate identification was not possible.

Because one pitfall trap in four different marshes was flooded during spring, we decided to randomly omit one trap from a total of five marshes in which 12 traps were available during spring; thus, we analyzed data from a total of 23 traps in each marsh.

### 2.3 Explanatory variables

#### 2.3.1 Weather data

For each sampled marsh, six variables were directly obtained or indirectly calculated from the nearest weather station (National Agricultural Service; http://agromet.inia.cl/estaciones.php): seasonal mean temperature (°C), maximum and minimum temperature of the season, coefficient of variation of temperature (°C), seasonal mean precipitation, and coefficient of variation of precipitation (mm). Coefficients of variation were calculated to quantify the potential of seasonal variability in temperature and precipitation on community assembly.

#### 2.3.2 Vegetation and edaphic data

In each marsh on each sampling occasion, six vegetation samples were taken randomly within the high marsh zone by harvesting 10‐cm^2^ circular plots at ground level. Plants were transported to the laboratory in plastic bags to prevent desiccations. Once in the laboratory, plants were transferred to a refrigerator before being processed. Material was processed within few days after collection. It was first separated into live and dead material and sorted into species, and then, it was dried at 60°C for 72 hr and weighed for specific biomass (i.e., the biomass of each plant species). We also measured the height of the tallest branch/ramet of each plot sample with a measuring stick and water content, which was expressed as the percent difference between initial and final weight of plants.

During the same visits, we also collected soil samples (ca. 10 cm large × 15 cm depth) in six locations randomly distributed within the high marsh zone. Sampling was conducted during periods of the lowest tides in the month to avoid the effect of seawater inundation that occasionally can occur, even within the high marsh zone. Samples were put in plastic bags and transported to the laboratory for analyses of soil water, organic matter content, and soil salinity. We measured water content gravimetrically after weighing, drying (for 48 hr at 80°C), and then reweighing each soil sample to the nearest 0.001 g using a Precisa XB 320 M balance (Precisa Gravimetrics, Switzerland). Water content was then expressed as the percent difference between initial and final weight of the soil sample. Organic matter was determined for the previously dried samples, which were combusted in a muffle at 500°C for 12 hr. Organic matter was then expressed as the percent difference in weight between dried samples before and after combustion. Soil pore water salinity was measured (six replicates per zone) by rehydrating dried soil samples in a known volume of distilled water, mixing constantly for 48 hr, and measuring the salinity of the resulting supernatant. We then calculated soil salinity gravimetrically as the difference between the original water content and the salinity of the supernatant. Measurements were made with a refractometer (±1%).

#### 2.3.3 Spatial data

These data included the terms of a third‐degree polynomial function of the geographic coordinates *X* and *Y* (nine terms: the centered site coordinates, *x *and *y*, and *x*2, *y*2, *xy*, *x*3, *y*3, *x*2*y*, and *xy*2) (Borcard, Legendre, & Drapeau, 1992). The *X* (latitude) and *Y* (longitude) terms describe linear spatial patterns in data, whereas the higher order term models indicated more complex landscape features such as gaps or patches (Borcard et al., 1992). Thus, these terms ensure the detection of more complex spatial features in the species data set than that provided by linear gradient patterns alone (Borcard et al., 1992), allowing the detection of broad‐scale spatial patterns of diversity measures. Particularly, this method is well suited to model among group structure of group of sites that are far from another in the map (Bocard, Gillet, & Legendre, 2018).

### 2.4 Statistical analysis

All analyses were conducted in the statistical programming environment R version 3.5.1 (R Development Core Team, 2018), including functions in the Vegan (ANOVA; hclust; ordiR2step; rda; vif.cca; var.part), Betapart (beta.multi; beta.pair), Ape (pcoa), Clustsig (simprof), labdsv (IndVal), and iNext (iNEXT) packages.

#### 2.4.1 Diversity patterns and sampling completeness

To evaluate the inventory completeness of each marsh, we calculated their sample coverage using the function *iNEXT *(Hsieh, Ma, & Chao, 2016). Sample coverage refers to the proportion of the total number of individual in a community that belongs to the species detected in the sample (Hsieh et al., 2016).

Analyses were based on abundance data and were repeated for the total community (i.e., family level, hereafter arthropods) and for Crustacea, Araneae, and Coleoptera (genus/species/morph species level, hereafter species) separately. Since we used the lowest possible taxonomic level (genus, species, or morph species), estimations were conservative.

#### 2.4.2 Similarity in community composition between salt marshes

To determine the degree of similarity in community composition between marshes, we used a hierarchical cluster analysis based on Hellinger distances (Legendre & Gallagher, 2001). Cluster analyses were performed using Ward linkage combined with a similarity profile permutation analysis (SIMPROF) to test the statistical significance of the clusters. When SIMPROF revealed significant clusters, we first repeated the analyses after excluding those taxa found only at one site, to evaluate their potential influence, and then, we calculated the indicator taxa of each of cluster using the indicator value method (IndVal) proposed by Dufrêne and Legendre (1997). The statistical significance of the indicator species was tested using 9,999 permutations. In the cases that SIMPROF analyses did not detect any significant clusters, we simply noted whether sites geographically close to each other clustered together. To investigate whether the similarity in community composition between marshes was related to those of environmental similarity, we used a cluster analyses as above. We used Euclidean distances of log (*x* + 1) or arcsine‐transformed variables. However, not all local data were available for all marshes in both seasons; thus, analyses were repeated for each season separately and on the averaged data, after excluding the marshes with incomplete sampling.

#### 2.4.3 Relative importance of turnover and nestedness in multiple‐marsh dissimilarity

To examine whether the overall multiple‐site dissimilarity of the arthropod community across all marshes (i.e., overall spatial differentiation) is structured mainly by spatial turnover or nestedness, we partitioned beta diversity into two components following Baselga (2010) as: *β*_sor_ = *β*_sim_ + *β*_nes_. *β*_sor_ is the Sorensen dissimilarity and represents the total difference in species composition between two sites, *β*_sim_ is the Simpson dissimilarity, which indicates species replacement by others from one site to another (turnover), and *β*_nes_ is the nestedness‐driven dissimilarity, which indicates the differences in the taxa collected per site due to the loss of taxa (nestedness). Analyses were conducted on the presence and absence families for the total community and on species/morphospecies for each group using the “beta.multi” function (Baselga, Orme, Villeger, De Bortoli, & Maintainer, 2017). Since differences in beta diversity between taxa are affected by the size of the regional species pool (Kraft et al., 2011), we did not compare differences among the studied groups.

#### 2.4.4 Factors affecting total beta diversity and its components

To estimate the relative contribution of spatial, climate, and local factors (i.e., soil and vegetation) to the overall beta diversity (*β*_sor_) and to its two components of turnover (*β*_sim_) and nestedness (*β*_nes_), we used redundancy analyses (RDAs) followed by variation partitioning (Borcard et al., 1992). First, pairwise measures of turnover (*β*_sim_), nestedness‐resultant dissimilarity (*β*_nes_), and overall beta diversity (*β*_sor_) between marshes were calculated using the function “beta.pair.” Then, PCoA analyses with Lingoes correction for negative eigenvalues (Legendre, 2014) were performed separately on the three pairwise beta diversity matrices using the function “pcoa” (Paradis, Claude, & Strimmer, 2004). Finally, the eigenvectors extracted from principal coordinate analysis (PCoA) were used as response variables in separate variation partitioning analyses.

To test the effects of spatial, climate, and local factors on the overall beta diversity and on its turnover and nestedness components, we performed separate RDAs on each data set (see Supporting Information Appendix S1 for regional and local variables). If the global test including all the explanatory variables of a data set was statistically significant (Supporting Information Appendix S2), a forward selection was then performed using the “ordiR2step” function. Forward selection was conducted with two stopping criterion: either *p* > 0.05 or the adjusted *R* ^2^ values of the reduced model exceeding those of the global model. Thus, only the variables that best explained the variability of each data set were retained.

The climate and edaphic data set included log (*x* + 1) or arcsine‐transformed variables, while the vegetation data set included the first three axes extracted from a principal component analysis (PCA) performed on vegetation data to reduce the number of variable and their multicollinearity. We used variation inflation factor (VIF) (“vif.cca” function) to evaluate the collinearity among the selected explanatory variables (VIF < 10).

Redundancy analysis models were constructed separately for the total community and for each group. Using variation partitioning (Borcard et al., 1992) on RDA models, we divided the total percentage of variation into shared and exclusive fractions of the different components (spatial, climate, edaphic, and vegetation) based on their adjusted coefficients of determination (*R* ^2^) (Peres‐Neto, Legendre, Dray, & Borcard, 2006).

Statistical significance of the full model and the unique contributions of the three sets of predictors were analyzed using the “ANOVA” function in vegan by means of a permutation test (maximum permutation = 200). Since edaphic or vegetation data were not available for all marshes (Supporting Information Appendix S1), we decided to remove the incomplete marshes before analyses.

Data from all the traps collected during the two seasons within a salt marsh were pooled before analyses, except for the last set of analyses in which data were not pooled across seasons to improve sample size.

## 3 RESULTS

### 3.1 Diversity patterns and inventory completeness

We identified a total of 105 taxa (51 identified to species/morphospecies level) belonging to 68 families dominated by insects in the nine marshes (Supporting Information Appendix S3). Isopoda (Crustacea) alone comprised 80% of all individuals collected (Supporting Information Appendix S3). Of the 68 families, 28 (41%) were found at only one marsh and two (3%; Lycosidae and Carabidae) were common across all marshes. Of the most abundant groups, 2 taxa (20%) of Crustacea, 12 taxa (50%) of Araneae, and 30 taxa (62.5%) of Coleoptera were found at only one marsh, while we did not find common taxa across all marshes for these groups.

Observed richness varied in each marsh from 13 to 29 for the whole community (i.e., family richness), from 3 to 6 for Crustacea, from 2 to 16 for Coleoptera, and from 3 to 9 for Aranea (Table 1).

**Table 1:** Mean (±*SE*) abundance, observed taxonomic richness, including sample completeness, across marshes for the total arthropod community (family level), Crustacea, Coleoptera, and Araneae (species/morphospecies level). Values refer to a total of 23 pit fall traps collected during spring and autumn 2016

	Salinas	Carrizal	Litre	Pachingo	Conchali	Pullally	Yali	Carampangue	Putemun
Total arthropods									
Mean abundance (±*SE*)	4.9 (1.14)	86.8 (10.3)	116.2 (28.0)	69.0 (18.3)	38.8 (8.8)	260.0 (40.5)	226.8 (37.4)	25.3 (5.4)	5.5 (1.3)
Observed richness	13	17	23	25	29	19	19	25	22
Sample coverage	0.928	0.998	0.997	0.995	0.986	0.999	0.998	0.984	0.937
Crustacea									
Mean abundance (±*SE*)	2.7 (0.8)	71.9 (9.9)	109.3 (26.6)	63.0 (17.5)	22.6 (7.8)	254.4 (40.2)	218.8 (37.0)	17.3 (4.4)	1.56 (0.55)
Observed richness	3	4	6	6	3	5	5	5	3
Sample coverage	1	1	1	1	1	1	1	1	1
Coleoptera									
Mean abundance (±*SE*)	1.26 (0.47)	5.5 (1.2)	5.4 (1.4)	2.0 (0.6)	7.9 (1.9)	2.8 (0.8)	4.6 (0.9)	2.3 (1.1)	0.8 (0.1)
Observed richness	2	5	8	9	16	9	11	15	13
Sample coverage	1	1	0.981	0.894	0.956	0.953	0.953	0.893	0.519
Araneae									
Mean abundance (±*SE*)	0.6 (0.1)	1.1 (0.3)	0.7 (0.2)	0.8 (0.3)	1.9 (0.5)	0.7 (0.2)	1.8 (0.3)	2.3 (0.9)	1.3 (0.33)
Observed richness	5	4	6	3	8	9	6	7	6
Sample coverage	0.745	0.964	0.760	1	0.861	0.661	0.929	0.926	0.935

Sample coverage was very high (often nearly 1.0) in all marshes for total arthropods and Crustacea (Table 1), suggesting that the inventory was equally complete among sites. For Coleoptera and Araneae, sample coverage varied between sites, but values were often above 75% (Table 1). Therefore, we considered it as fairly complete (Kaltsas, Trichas, Kougioumoutzis, & Chatzaki, 2013; Meijer, Whittaker, & Borges, 2011).

### 3.2 Similarity in community composition between marshes

**Figure 2:**
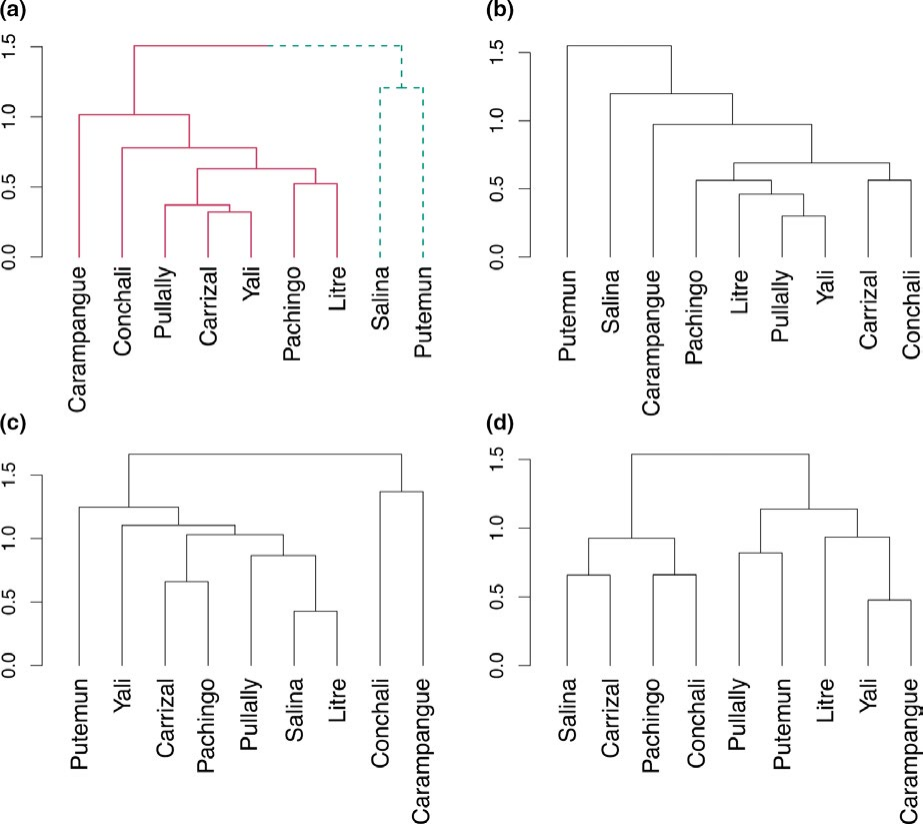
Cluster analyses based on the total arthropods (a), Crustacea (b), Coleoptera (c), and Araneae (d) abundances showing the degree of similarity between marshes. Different colors indicate significant different clusters according to SIMPROF analyses. Significant clusters are only those in (a)

The cluster analyses based on abundance data showed two major groups of clusters composed of different marshes for each taxon (Figure 2). Nonetheless, SIMPROF test (*p* < 0.05) only validated those of the whole arthropod community (Figure 2a). The first cluster included the two most extreme marshes (Salinas, hyperarid region; and Putemun, hyper‐humid region) and the second one the remaining seven marshes. Similar results were obtained after excluding the rare families. Indicator species analyses showed that only three families were significantly associated with these groups. The families Gnaphosidae (Araneae) and Nabidae (Hemiptera) were characteristic of group 1 (Indicator Values Index [IVI] = 0.92; 0.93, respectively; *p* < 0.05). The family Philoscidae (Crustacea) was characteristic of group 2 (IVI = 0.80; *p* = 0.026).

For each taxon, sites located close to each other were not more similar (Figure 2b–d). Instead, communities in marshes among different climatic regions (sensu Santibañez & Santibañez, 2008) were often more similar than marshes within the same climatic region. When considering the environmental similarity among marshes, cluster analyses followed by the SIMPROF test showed different groups among marshes respect to those emerged from the community similarity analyses (Supporting Information Appendix S4 and Figure 2).

### 3.3 Multiple‐marsh dissimilarities

**Figure 3:**
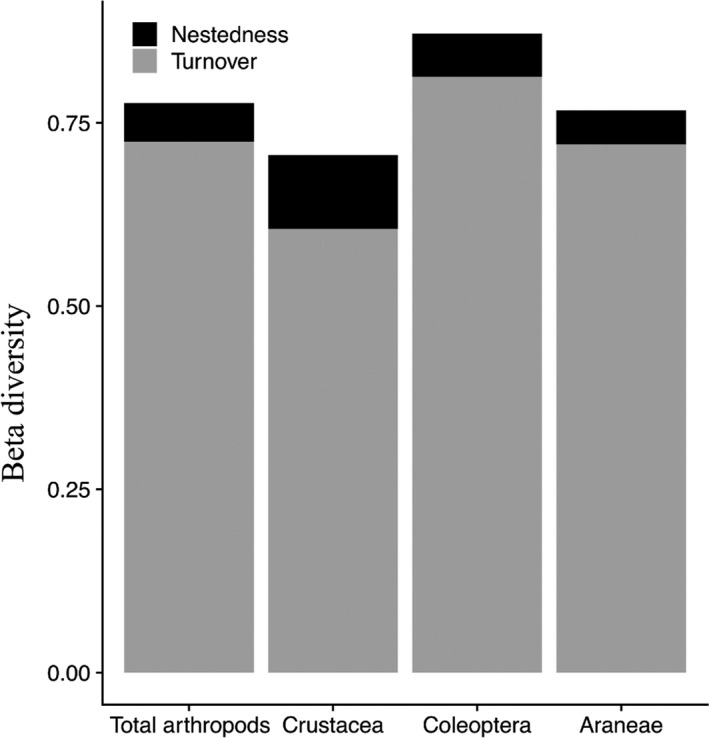
Partitioning of Sorenson beta diversity into the turnover and nestedness components of the total arthropods, Crustacea, Coleoptera, and Araneae across all nine marshes (multiple‐site dissimilarity)

Total beta diversity (*β*_sor_ multiple‐site dissimilarity) ranged from 0.71 for Crustacea to 0.87 for Coleoptera (Figure 3). For each taxon, community composition variation was explained mainly by species turnover (*β*_sim_; species replacement between marshes), rather than nestedness (*β*_nes_; species loss from one marsh to another) with values ranging from 0.60 to 0.81 and from 0.05 to 0.1 (Figure 3). Although weak, the nestedness component of beta diversity was highest in Crustacea compared to the other groups.

### 3.4 Drivers of total beta diversity and its turnover component

For total arthropods, the selected model, which includes space, climate, vegetation, and edaphic characteristics (Table 2), explained 23.3% (adjusted *R* ^2^) of the beta diversity variation (*p* = 0.001; *F* = 1.9126). The space, climate, vegetation, and edaphic variable explained similar proportion of variation (global fraction between 0.10 and 0.12, see Supporting Information Appendix S5).

**Table 2:** Subset of variables selected from the forward selection procedure from the spatial, climate, edaphic, and vegetation data sets (*n* = 16) explaining total arthropod, Crustacea, Coleoptera, and Araneae beta diversity (*β*_sor_) and its turnover component (*β*_sim_)

	Spatial	Climate	Edaphic	Vegetation
Total arthropods				
*β*_sor_	*Y* (0.05)	*T*_Season_ (0.12)	Water soil (0.11)	PC1veg (0.05) PC2veg (0.10)
*β*_sim_	*Y* (0.21) *Y*2 (0.13) *Y*3 (0.16)	*T*_Season_ (0.06)	GNS	PC1veg (0.06)
Crustacea				
*β*_sor_	*Y* (0.13)	P (0.08) *T*_min_ (0.13)	Water soil (0.08)	GNS
*β*_sim_	*Y* (0.07)	GNS	GNS	GNS
Araneae				
*β*_sor_	NS	GNS	GNS	NS
*β*_sim_	*Y*2 (0.02)	GNS	GNS	NS
Coleoptera				
*β*_sor_	*Y* (0.13) *Y*2 (0.18)	GNS	Salinity (0.11)	GNS
*β*_sim_	*Y* (0.08)	GNS	Salinity (0.07)	GNS

Note

GNS: global model not significant; NS: not‐selected after forward selection; PC1 veg: PC scores of the first PCs extracted to summarize vegetation variables; PC2 veg: PC scores of the second PCs extracted to summarize vegetation variables; *T*_min_: minimum temperature of the sampled season; *T*_Season_: mean temperature of the sampled season; *Y*: longitude; *Y*2: quadratic polynomial trend surface of longitude; *Y*3: cubic polynomial trend surface of longitude.

Values indicate adjusted (*R* ^2^). All selected variables are statistically significant.

**Figure 4:**
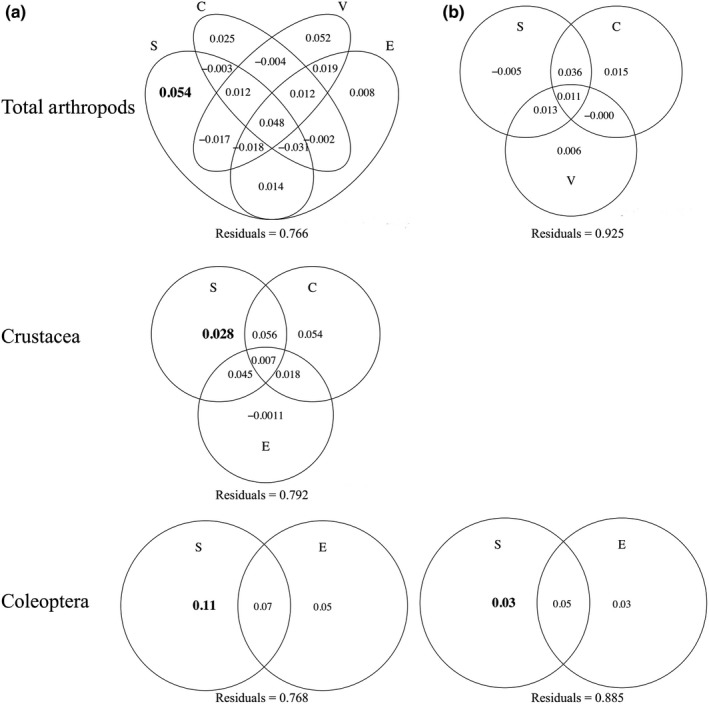
Pure and shared effect of spatial variables (S), weather variables (C), edaphic variables (E), vegetation variables (V), and their joint effects on (a) beta diversity (*β*_sor_) and (b) turnover (*β*_sim_) component of the studied taxon (*n* = 16). Values indicate the proportion of variance (adjusted *R* ^2^). Significant fractions are in bold

Variation partitioning showed that space alone explained significantly more of the variance of the overall beta diversity (5.4%, Figure 4a) than climate alone (2.5%), edaphic variables alone (0.8%), and vegetation alone (5.2%), whose contributions were not significant (*p* > 0.05). All the shared fractions among climate, soil, and vegetation explained small proportions of variation (4.8%, Figure 4a). Space, climate, and vegetation (Table 2) were able to explain only 7.4% of the variation in species turnover for total arthropods (*p* = 0.004; *F* = 1.2405). Climate and vegetation alone explained small, not significant (*p* > 0.05) variation in family turnover (1.5% and 0.6%, respectively, see Figure 4b). The joint effect of space and climate explained more variation in arthropod turnover (3.6%, see Figure 4b) compared to those of climate and vegetation (1%) and to those of space, climate, and vegetation together (1.1%).

For Crustacea, the selected model, which includes spatial, climate, and edaphic variables, explained 20.8% of the variation in beta diversity (*p* = 0.001; *F* = 1.9837). Variance partitioning revealed that space alone accounted for a small but significant proportion (2.8%) of Crustacea beta diversity, whereas the exclusive contribution of climate and water content in soil was not significant (*p* > 0.05). The joint effect of space, climate, and soil explained only 0.7% of the Crustacea beta diversity (Figure 4a). Only space was a significant predictor of Crustacea turnover, explaining 7.2% (*p* = 0.001; *F* = 2.173).

For Coleoptera, the selected model, which includes space and soil salinity, explained 23.1% of the overall variation (*p* = 0.002; *F* = 2.4023). Space alone accounted for a significant proportion of the total variation (11.2%), whereas the joint effect of space and soil explained the 7.0% (Figure 4a).

Space and soil salinity explained the 11.4% of Coleoptera turnover (*F* = 1.907; *p* = 0.003). Space alone accounted for a small, but significant proportion of variation (3.0%), whereas the joint effect between them explained the 5.0% of the species turnover (Figure 4b).

For Aranea, we did not find any significant variable explaining overall beta diversity, whereas spatial effects affected their turnover (7.3%; *F* = 2.1033; *p* = 0.002).

Given the limited contribution of nestedness to arthropod geographic diversity, and because almost no factor was found to explain the differences in nestedness across marshes, we decide to not consider factors affecting nestedness further.

## 4 DISCUSSION

To our knowledge, this is the first study that partitions the beta diversity of salt marsh arthropods into its turnover and nestedness components in a long geographic latitudinal gradient and partitioned the relative influence of regional (e.g., climate), spatial, and local environmental variables (e.g., soil and vegetation) on patterns of beta diversity over a large scale. We showed that spatial variables and spatially structured environmental variables influenced the assembly of the entire arthropod community and the most abundant and speciose taxa, but their importance was different among the studied groups. Although our findings are based on a small data set and thus are not definitive, this study represents a first step in understanding what drives patterns of biodiversity in a poorly understood ecosystem.

### 4.1 Potential geographic clustering

Our results did not support the prediction that communities between closer marshes are more similar to each other than those between distant marshes, because they possess similar climate and environmental conditions. Instead, arthropod assemblage at the family level clearly separated into two groups with the two most distant marshes of Salinas and Putemun grouped together (Figure 2). While the decrease in community similarity with increasing distance between two sampled sites has been consistently shown in biogeographic studies (distance decay; Jobe, 2008; Nekola & White, 1999; Soininen, McDonald, & Hillebrand, 2007), high similarity over long distance has also been reported for several taxa, including invertebrates (Antonini et al., 2017; Condit et al., 2002; Vasconcelos, Vilhena, Facure, & Albernaz, 2010). However, the high similarity found in this study can be the result of incomplete sampling and the omission of rare taxa. Nonetheless, we obtained similar results after excluding the families found at only one site, a finding in line with Hulcr, Novotny, Maurer, and Cognato (2008) who demonstrated that including or excluding rare taxa did not affect the similarity between sites over long distances. Thus, the high similarity between Salinas and Putemun is most likely because some arthropod families are common at both sites (Supporting Information Appendix S3). Specifically, the Araneae belonging to the family Gnaphosidae and the Hemiptera belonging to the family Nabidae were the most common taxa in both the Salinas and Putemun marshes. Individuals in both families are xerophilic or are able to tolerate high aridity and saline soil as well as temperature stress (Chatzaki, Lymberakis, Markakis, & Mylonas, 2005; Yin et al., 2017). Although soil salinities in Salinas and Putemun are very high compared to the other marshes (Supporting Information Appendix S1), we did not find any strong salinity effect on total arthropods. Thus, it seems possible that historical events might have influenced the present distribution. For example, the increased hyperarid conditions of the Atacama region that occurred during the Quaternary (Fernández et al., 2016; Latorre et al., 2007) may have affected the distribution of the families of Nabidae and Gnaphosidae, favoring similarity in arthropod composition even over large distances.

As a whole, these results suggest that there is not a unique arthropod community in each bioclimatic region.

### 4.2 Drivers of multiple‐site dissimilarities

The total arthropod community and each studied group showed high beta diversity values among all studied marshes in Chile (Figure 3). Species turnover contributed much more to the high beta diversity than nestedness for all groups, which supports our predictions and is in line with other studies comparing taxa with different dispersal abilities (Viana et al., 2016; Zellweger, Roth, Bugmann, & Bollmann, 2017). The dissimilarities between salt marshes were thus driven by differences in community composition rather than by differences in family or species richness (Viana et al., 2016). This in turn suggests that each marsh has the capacity to accommodate unique invertebrate taxa, underlying their values for the biodiversity conservation of arthropods.

Nonetheless, we cannot rule out the possibility that the very low nestedness values reported here were the result of the high number of un‐sampled marshes along the Chilean coast, which are numerous and highly connected (Marquet, Abades, & Barría, 2017). Further studies will be needed to address this in the future, for example by adding additional sites.

### 4.3 Processes affecting beta diversity

Beta diversity of total arthropod, Crustacean, and Coleoptera along the Chilean coast was mainly spatially structured. That is, each group distributes along the coast depending on the different regional characteristics of the studied sites.

Arthropod replacement from one site to other (turnover) was related primarily to the spatially structured variation of climate, whereas Coleoptera turnover was related mainly to those of edaphic factors (i.e., salinity).

The importance of climate in controlling the geographic range of species has been long recognized (Grinnell, 1914), and climate has been also frequently considered a good predictor of arthropod variation (Jiménez‐Valverde, Baselga, Melic, & Txasko, 2010; Lewthwaite, Debinski, & Kerr, 2017). Similarly, salinity may affect arthropods either physiologically or behaviorally by altering their osmoregulatory processes or influencing their habitat choice (Pétillon, Lambeets, Ract‐Madoux, Vernon, & Renault, 2011). Coastal wetlands along the Chilean coast provide very different temperatures and soil salinity conditions to their inhabitants (Supporting Information Appendix S1). Since total arthropods in our study include ecologically distinct families with a broad range of environmental requirements (food, habitat, and climatic preferences), and Coleoptera may exhibit different tolerance to salinity (Pétillon et al., 2008), the climatic and edaphic differences across marshes may thus result in a gradient that filters families (i.e., families replace each other) or species according to their dependence (physiological or behavioral) on specific climate or saline soil.

These results reinforce the findings of previous studies demonstrating that environmental filtering reveals turnover in several taxa including invertebrates over a large scale (Alahuhta et al., 2017; Viana et al., 2016; Zellweger et al., 2017).

The fact that we also found higher order terms affecting total arthropod and Coleoptera distribution suggests that more complex landscape patterns can also explain their large‐scale variation. For example, climate and salinity affect plant growth and productivity, which in turn affects resource availability (resource availability hypothesis sensu Coley, Bryant, & Chapin, 1985). Thus, more potentially structured variables should be included in future studies.

We also showed that space alone explained significant more variation than climate and local variables for total arthropod and Coleoptera beta diversity and for Crustacean and Araneae turnover. However, spatial variation for total arthropod and Crustacean was mainly related to longitude, whereas for Araneae to the quadratic term of longitude, which also influenced Coleoptera beta diversity (Table 2).

The effect of linear spatial distance, which is a proxy of dispersal limitation and colonization, suggests that the assembly of total arthropod and Crustacea along the Chilean coast can be also driven by dispersal‐based processes. For both groups, current beta diversity could be related to dispersal constraints that happened in historical time. For example, species turnover across Chilean marshes could have been associated with (a) incomplete re‐colonization following the well‐known Chilean Quaternary glacial history (Latorre et al., 2007), which may have produced species replacement due to speciation and extinction events, or (b) differences in stochastic colonization events among vacant patches when they became available in the past. This may be because early colonizers prevented late‐arriving species from establishing in the community (Urban & De Meester, 2009). Nonetheless, for Crustacea we cannot rule out the possibility that their present diversity could be related to their poor dispersal ability in ecological time (i.e., contemporary), because they should be most influenced by the isolation by distance between marshes. Alternatively, the significant effect of space could be also the result of unmeasured environmental variables that were spatially structured (Dray et al., 2012). Although we used some of the variables that are best known to affect terrestrial arthropod distribution, other factors (e.g., nutrient content of plants, human influence, or pathogens) may also have a role. Future studies are required to identify other potential factors that could influence the variation of arthropod beta diversity over a large scale.

For Araneae and for Coleoptera, the spatial variation of their turnover and beta diversity indicated the effect of more complex configurations between the studied marshes that affect their distribution (Legendre & Legendre, 1998), for example, the presence of barriers or obstacles (e.g., structure of the landscape) to their dispersal (Bell, Bohan, Shaw, & Weyman, 2005), but also biotic interactions, which has been shown to be important in determining large‐scale distribution patterns (Wisz et al., 2013).

Taken together, our results did not support our expectation that arthropod assemblages along the Chilean coast are driven mainly by the local vegetation. Instead, we showed that the geographic patterns of arthropod beta diversity in Chile are largely the result of regional factors and present/past colonization dynamics, and that present local conditions (salinity) can also play a role.

Specifically, dispersal‐based process might have determined the initial composition of total arthropod and Crustacea, and species sorting mediated by the environmental variation between marshes might have maintained their assemblages. Instead, species sorting driven by the variation of salinity among marshes primarily determined the distribution of Coleoptera along the coast. For Araneae, while we suggested that historical factors might have had a role in explaining family distribution, we are unable to assess the factors and mechanisms affecting their large‐scale distribution at a finer taxonomic resolution.

Nonetheless, it should be noted that a significant proportion of the variation for the whole community and for each group was undetermined. This could be partly because coastal wetlands face frequent natural disturbances (i.e., are highly dynamic) that can result in stochastic distributions, but also deterministic variation caused by unmeasured environmental variables. Further studies will be needed to clarify this in the future

## 5 CONCLUSION

Arthropod assemblages of salt marshes along the coast of Chile are structured by a combination of large‐scale (climate and spatial) and local variables (edaphic), and the effect of these variables is different for each group. In these terms, it is important to consider the taxonomic resolution and the differences in the ecological requirements among taxa to explain large‐scale diversity patterns.

The predominance of turnover for arthropods in the studied marshes and the high number of rare taxa (here referred as those occurring only at one site) indicated that arthropod communities are very different to each other, pointing toward the benefit of maintaining wetland variation along the Chilean coast to conserve arthropod biodiversity.

Further studies will be needed to address this in the future and to evaluate the functional significance of arthropod in the Chilean coastal wetlands.

## CONFLICT OF INTEREST

None declared.

## AUTHOR CONTRIBUTIONS

CC and JMF conceived the ideas; CC and JMF collected the data; CC analyzed the data; and CC led the writing.

## Data Availability

All data supporting this study are openly available from Dryad https://doi.org/10.5061/dryad.h2f3t2n.
