# Supplementary material for: Partitioning the effects of regional, spatial, and local variables on beta diversity of salt marsh arthropods in Chile
Source: Ecol Evol. 2019 Jan 30;9(5):2575–87. doi: 10.1002/ece3.4922 (PMC6405494; doi:10.1002/ece3.4922)
Supplement: Supplementary file 1 [file ECE3-9-2575-s001.docx]

**Appendix 1**. Mean (± SE) values of climate, edaphic and vegetation variables collected during autumn and spring 2016 and used in the RDA and forward selection analyses.

Asterisks indicate the marshes not included in the analyses because some variables were missed during one sampling event.

|  | Salina | Carrizal | Litre | Pachingo | Conchali | Pullally* | Yali | Carampange | Putemun* |
| --- | --- | --- | --- | --- | --- | --- | --- | --- | --- |
| **Autumn** | | | | | | | | | |
| **Climate** |  |  |  |  |  |  |  |  |  |
| Temperature (ºC) | 17  (0.95) | 13.9  (1.14) | 15.05  (1.09) | 15.05  (1.09) | 13.63  (1.45) | 13.90  (1.53) | 12.35  (1.98) | 12.38  (1.15) | 10.18  (1.13) |
| Precipitation (mm) | 1.3  (0.67) | 2.98  (0.78) | 10.78  (5.35) | 10.78  (5.35) | 30.65  (15.40) | 53.33  (25.01) | 51.25  (28.50) | 42.85  (21.50) | 66.70  (21.83) |
| CVT | 0.11 | 0.16 | 0.15 | 0.15 | 0.21 | 0.22 | 0.32 | 0.19 | 0.22 |
| CVP | 1.03 | 0.53 | 0.99 | 0.99 | 1.01 | 0.94 | 1.11 | 1.00 | 0.65 |
| Tmin | 2.13  (0.87) | -8.15  (10.57) | 6.78  (1.41) | 6.78  (1.41) | 3.88  (1.44) | 5.30  (1.30) | 1.58  (1.38) | 6.08  (1.28) | 1.30  (1.87) |
| Tmax | 33  (0.82) | 27.8  (0.59) | 29.05  (1.61) | 29.05  (1.61) | 28.85  (1.78) | 28.70  (1.70) | 25.78  (3.13) | 21.00  (2.10) | 18.38  (1.70) |
| **Edaphic** |  |  |  |  |  |  |  |  |  |
| Organic Matter(%) | 2.63  (0.15) | 1.01  (0.22) | 1.38  (0.45) | 0.46  (0.05) | 2.32  (0.41) | 0.71  (0.16) | 2.30  (0.42) | 2.84  (0.39) | 6.07  (0.25) |
| Water Soil (%) | 15.20  (0.29) | 26.32  (1.31) | 19.22  (1.22) | 11.37  (1.61) | 20.98  (3.43) | 19.08  (2.15) | 20.04  (2.22) | 37.88  (1.98) | 78.57  (1.52) |
| Salinity | 12.3  (0.83) | 3.21  (0.99) | 4.74  (1.72) | 0.94  (0.08) | 0.23  (0.46) | 0.92  (0.16) | 0.60  (0.13) | 0.55  (0.18) | 13.77  (1.27) |
| **Vegetation** |  |  |  |  |  |  |  |  |  |
| ***Spartina*** |  |  |  |  |  |  |  |  |  |
| Live Height (cm) | 0 | 0 | 0 | 0 | 0 | NA | 67.12  (4.79) | 51.38  (2.85) | 42.22  (2.97) |
| Dry weight | 0 | 0 | 0 | 0 | 0 | NA | 21.58  (1.44) | 8.97  (1.14) | 6.63  (1.06) |
| Humidity (%) | 0 | 0 | 0 | 0 | 0 | NA | 47.82  (2.98) | 67.09  (0.64) | 67.93  (1.97) |
| Tot live weight (%) | 0 | 0 | 0 | 0 | 0 | NA | 63.50  (7.32) | 84.13  (2.82) | 73.03  (3.72) |
| Tot Fresh Weight | 0 | 0 | 0 | 0 | 0 | NA | 41.61  (2.30) | 27.35  (3.52) | 21.26  (3.74) |
| ***Distichilis*** |  |  |  |  |  |  |  |  |  |
| Live Height (cm) | 17.62  (5.90) | 27.83  (4.21) | 0 | 15.55  (7.39) | 29.06  (4.74) | 0 | 0 | 0 | 24.72  (2.20) |
| Dry weight  (gr) | 7.38  (2.81) | 7.59  83.40) | 0 | 3.68  (1.67) | 5.62  (0.74) | 0 | 0 | 0 | 1.01  (0.21) |
| Humidity (%) | 41.35  (12.69) | 53.24  (8.13) | 0 | 17.59  (7.90) | 41.79  (4.90) | 0 | 0 | 0 | 70.03  (9.00) |
| Tot live weight (%) | 61.75  (19.54) | 96.76  (2.39) |  | 21.01  (11.29) | 55.97  (12.85) |  |  |  | 100  (0) |
| Tot Fresh Weight (gr) | 16.69  4.88) | 14.30  (5.73) | 0 | 5.66  (2.55) | 10.37  (1.51) | 0 | 0 | 0 | 3.71  (0.45) |
| ***Sarcocornia*** |  |  |  |  |  |  |  |  |  |
| Live Height (cm) | 24.35  (10.95) | 30.13  (9.9) | 53.48  (8.33) | 37.63  (2.68) | 42.78  (12.92) | NA | 46.00  (4.84) | 0 | 0 |
| Dry weight  (gr) | 15.69  (7.65) | 17.91  (6.80) | 60.35  (4.70) | 47.67  (1.20) | 24.40  (6.70) | NA | 26.79  (2.85) | 0 | 0 |
| Humidity (%) | 27.97  (13.00) | 51.72  (16.73) | 62.90  (6.23) | 79.81  (1.56) | 43.68  (10.05) | NA | 81.28  (2.31) | 0 | 0 |
| Tot live weight (%) | 46.88  (21.14) | 66.52  (21.03) | 76.77  (15.60) | 97.00  (1.56) | 59.57  (16.36) | NA | 98.85  (0.90) | 0 | 0 |
| Tot Fresh Weight  (gr) | 37.53  (18.41) | 82.51  (27.95) | 178.35  (21.39) | 245.78 | 59.09  (9.10) | Na | 149.33  (16.27) | 0 | 0 |
|  |  |  |  |  |  |  |  |  |  |
| **Spring** | | | | | | | | | |
| **Climate** |  |  |  |  |  |  |  |  |  |
| Temperature (ºC) | 18.55  (0.87) | 15.50  (1.12) | 15.93  (1.16) | 15.93  (1.16) | 15.33  (1.30) | 16.20  (1.30) | 15.18  (1.47) | 13.35  (0.78) | 10.93  (0.93) |
| Precipitation (mm) | 1.45  (1.23) | 0.85  (0.43) | 3.48  (1.97) | 3.48  (1.97) | 6.75  (4.10) | 7.90  (5.18) | 15.15  (7.74) | 45.08  (14.74) | 61.80  (11.17) |
| CVT | 0.09 | 0.14 | 0.15 | 0.15 | 0.17 | 0.16 | 0.19 | 0.12 | 0.17 |
| CVP | 1.69 | 1.00 | 1.14 | 1.14 | 1.22 | 1.31 | 1.02 | 0.65 | 0.36 |
| Tmin | 2.88  (0.67) | 6.65  (1.35) | 7.25  (1.78) | 7.25  (1.78) | 3.30  (1.98) | 4.75  (1.55) | 1.88  (1.90) | 4.18  (1.09) | 2.33  (1.54) |
| Tmax | 33.78  (0.35) | 29.48  (0.43) | 29.95  (0.53) | 29.95  (0.53) | 32.80  (0.93) | 35.03  (1.40) | 32.53  (2.17) | 26.00  (1.98) | 22.13  (1.25) |
| **Edaphic** |  |  |  |  |  |  |  |  |  |
| Organic Matter(%) | 2.38  (0.09) | 2.47  (0.55) | 1.24  (0.07) | 0.42  (0.04) | 1.69  (0.39) | 0.42  (0.14) | 0.75  (0.04) | 0.34  (0.01) | NA |
| Water Soil (%) | 24.38  (3.4) | 19.03  (2.35) | 14.38  (1.48) | 8.59  (1.02) | 25.40  (4.84) | 13.21  (0.17) | 26.30  (1.41) | 15.86  (0.33) | NA |
| Salinity | 16.27  (4.32) | 8.66  (3.16) | 10.02  (1.68) | 3.09  (0.69) | 0.79  (0.25) | 1.43  (0.10) | 1.63  (0.33) | 0.17  (0.01) | NA |
| **Vegetation** |  |  |  |  |  |  |  |  |  |
| ***Spartina*** |  |  |  |  |  |  |  |  |  |
| Live Height (cm) | 0 | 0 | 0 | 0 | 0 | 59.78  (3.79) | 68.95  (7.07) | 69.72  (3.86) | 43.80  (2.24) |
| Dry weight  (gr) | 0 | 0 | 0 | 0 | 0 | 33.87  (2.22) | 66.78  (5.54) | 18.57  (5.18) | 24.73  (3.69) |
| Humidity (%) | 0 | 0 | 0 | 0 | 0 | 37.14  (2.62) | 54.21  (1.04) | 61.84  (2.80) | 60.26  (1.95) |
| Tot live weight (%) | 0 | 0 | 0 | 0 | 0 | 37.77  (6.74) | 58.55  (4.92) | 86.39  (4.82) | 85.04  (5.40) |
| Tot Fresh Weight (gr) | 0 | 0 | 0 | 0 | 0 | 54.73  (5.21) | 145.16  (9.86) | 46.67  (11.80) | 61.31  (8.12) |
| ***Distichilis*** |  |  |  |  |  |  |  |  |  |
| Live Height (cm) | 0 | 53.23  (4.70) | 0 | NA | 0 | 0 | 0 | 0 | 0 |
| Dry weight (gr) | 0 | 15.51  (5.18) | 0 | 0.29  (0.24) | 0 | 0 | 0 | 0 | 0 |
| Humidity (%) | 0 | 48.56  (1.77) | 0 | 6.17  (4.78) | 0 | 0 | 0 | 0 | 0 |
| Tot live weight (%) | 0 | 90.12  (4.18) | 0 | NA | 0 | 0 | 0 | 0 | 0 |
| Tot Fresh Weight (gr) | 0 | 30.97  (10.5) | 0 | 0.37  (0.24) | 0 | 0 | 0 | 0 | 0 |
| ***Sarcocornia*** |  |  |  |  |  |  |  |  |  |
| Live Height (cm) | 56.97  (2.95) | 50.52  (3.7) | 42.57  (3.97) | 62.18  (6.14) | 52.60  (3.81) | 0 | 0 | 0 | 0 |
| Dry weight  (gr) | 94.54  (9.04) | 58.82  (7.9) | 57.92  (3.19) | 60.82  (5.06) | 52.06  (5.68) | 0 | 0 | 0 | 0 |
| Humidity (%) | 66.49  (0.97) | 74.10  (3.64) | 66.34  (1.82) | 69.83  (3.87) | 72.80  (2.29) | 0 | 0 | 0 | 0 |
| Tot live weight (%) | 97.26  0.75) | 96.14  (1.24) | 98.92  (0.55) | 89.56  (3.5) | 100.00  (0) | 0 | 0 | 0 | 0 |
| Tot Fresh Weight (gr) | 283.26  (29.38) | 230.63  (14.78) | 174.00  (12.39) | 212.77  (24.09) | 196.30  (21.96) | 0 | 0 | 0 | 0 |
