# Supplementary material for: Partitioning the effects of regional, spatial, and local variables on beta diversity of salt marsh arthropods in Chile
Source: Ecol Evol. 2019 Jan 30;9(5):2575–87. doi: 10.1002/ece3.4922 (PMC6405494; doi:10.1002/ece3.4922)
Supplement: Supplementary file 2 [file ECE3-9-2575-s002.docx]

| Taxon | Components of β diversity | Dataset | Variance | F | P |
| --- | --- | --- | --- | --- | --- |
| Tot  Arthropod | βsim | S | 0.39717 | 1.2047 | 0.004 |
|  | βnes |  | 0.17996 | 1.0108 | 0.557 |
|  | βsor |  | 0.188439 | 2.1116 | 0.001 |
|  | βsim | C | 0.27516 | 1.2075 | 0.016 |
|  | βnes |  | 0.11590 | 0.9513 | 0.707 |
|  | βsor |  | 0.12854 | 1.6148 | 0.002 |
|  | βsim | E | 0.12692 | 1.036 | 0.374 |
|  | βnes |  | 0.071268 | 1.2537 | 0.01 |
|  | βsor |  | 0.075134 | 1.7392 | 0.013 |
|  | βsim | V | 0.15445 | 1.3357 | 0.002 |
|  | βnes |  | 0.048338 | 0.7724 | 0.99 |
|  | βsor |  | 0.072915 | 1.6665 | 0.008 |
| Crustacea | βsim | S | 0.48706 | 1.1745 | 0.026 |
|  | βnes |  | 0.16870 | 1.1264 | 0.233 |
|  | βsor |  | 0.240176 | 1.9298 | 0.001 |
|  | βsim | C | 0.32183 | 1.0927 | 0.19 |
|  | βnes |  | 0.11473 | 1.119 | 0.18 |
|  | βsor |  | 0.15651 | 1.4088 | 0.04 |
|  | βsim | E | 0.16376 | 1.092 | 0.202 |
|  | βnes |  | 0.06955 | 1.398 | 0.033 |
|  | βsor |  | 0.09694 | 1.7142 | 0.011 |
|  | βsim | V | 0.16325 | 1.0877 | 0.225 |
|  | βnes |  | 0.055748 | 1.048 | 0.35 |
|  | βsor |  | 0.078335 | 1.2799 | 0.149 |
| Coleoptera | βsim | S | 0.58676 | 1.3563 | 0.006 |
|  | βnes |  | 0.42720 | 1.0523 | 0.368 |
|  | βsor |  | 0.29592 | 2.8768 | 0.001 |
|  | βsim | C | 0.36760 | 1.0667 | 0.299 |
|  | βnes |  | 0.29489 | 1.0987 | 0.177 |
|  | βsor |  | 0.17603 | 1.325 | 0.081 |
|  | βsim | E | 0.24342 | 1.5298 | 0.004 |
|  | βnes |  | 0.11830 | 0.8116 | 0.954 |
|  | βsor |  | 0.11418 | 17518 | 0.007 |
|  | βsim | V | 0.19875 | 1.1598 | 0.162 |
|  | βnes |  | 0.12167 | 0.8401 | 0.923 |
|  | βsor |  | 0.08441 | 1.1516 | 0.288 |
| Araneae | βsim | S | 0.45615 | 1.2716 | 0.035 |
|  | βnes |  | 0.119374 | 0.9169 | 0.69 |
|  | βsor |  | 0.28107 | 1.3788 | 0.068 |
|  | βsim | C | 0.31072 | 1.2018 | 0.087 |
|  | βnes |  | 0.066092 | 0.7016 | 0.982 |
|  | βsor |  | 0.18735 | 1.207 | 0.166 |
|  | βsim | E | 0.15030 | 1.091 | 0.267 |
|  | βnes |  | 0.038722 | 0.9281 | 0.614 |
|  | βsor |  | 0.09674 | 1.192 | 0.228 |
|  | βsim | V | 0.17885 | 1.376 | 0.024 |
|  | βnes |  | 0.040077 | 0.9691 | 0.505 |
|  | βsor |  | 0.12091 | 1.6214 | 0.022 |

**Appendix 2**. Results global test realized using RDA analyses on the spatial (S) dataset (X, X2, X3, Y, XY,X2Y,Y2,XY2,Y3), weather (C) data set [seasonal mean temperature (ºC), maximum and minimum temperature of the season, coefficient of variation of temperature (ºC), seasonal mean precipitation and coefficient of variation of precipitation (mm)], edaphic (E) dataset (organic matter, water content in the soil

and soil salinity ) and vegetation (V) dataset (PC1, PC2 and PC3).
