# Supplementary material for: Partitioning the effects of regional, spatial, and local variables on beta diversity of salt marsh arthropods in Chile
Source: Ecol Evol. 2019 Jan 30;9(5):2575–87. doi: 10.1002/ece3.4922 (PMC6405494; doi:10.1002/ece3.4922)
Supplement: Supplementary file 3 [file ECE3-9-2575-s003.docx]

**Appendix 3.** Sum of all taxa recorded in the 9 salt marshes during two seasons (*N* = 23).

|  | **Species/**  **genus/morfo** | **Salinas** | **Carrizal** | **Litre** | **Pachingo** | **Conchalí** | **Pullaly** | **Yali** | **Carampangue** | **Putemun** |
| --- | --- | --- | --- | --- | --- | --- | --- | --- | --- | --- |
| **Thysanura** |  |  |  |  |  |  |  |  |  |  |
| Machilidae |  |  |  |  |  |  |  |  | 60 |  |
| **Myriapoda** |  |  |  |  |  |  |  |  |  |  |
| Dalodesmidae |  |  |  |  |  |  |  |  |  | 20 |
| Cambalidae |  |  |  |  |  |  |  |  |  | 10 |
| **Mollusca** |  |  |  |  |  |  |  |  |  |  |
| Ellobiidae |  |  |  |  |  |  |  |  |  | 9 |
| Helicidade |  |  |  |  |  | 2 |  |  |  |  |
| **Amphipoda** |  |  |  |  |  |  |  |  |  |  |
| Talitridae | *Atacamorchestia* spp. |  | 247 |  |  |  |  |  |  |  |
| Talitridae | *Orchestia gammarellus* |  |  |  | 12 | 82 | 1 | 37 | 1 | 6 |
| Talitridae | *Transorchestia chiliensis* |  |  | 8 | 203 |  | 272 | 1094 | 87 | 28 |
| **Isopoda** |  |  |  |  |  |  |  |  |  |  |
| Detonidae | *Scyphoniscus* spp | 1 | 14 | 1 |  |  | 135 | 10 |  |  |
| Philoscidae | Benthanoides spp. | 16 | 1391 | 2298 | 866 | 438 | 5416 | 3890 | 153 |  |
| Halophiloscidae | *Halophiloscia couchii* |  |  | 99 | 25 |  |  |  |  |  |
| Platyartridae | *Niambia capensis* | 46 | 3 | 14 | 70 |  |  | 3 | 13 |  |
| Porcellionidae | *Porcelio scaber* |  |  |  |  | 1 |  |  | 144 |  |
| Styloniscidae | *Styloniscus* spp |  |  |  |  |  |  |  |  | 2 |
| Tylidae | *Tylos chilensis* |  |  | 92 | 274 |  | 27 |  |  |  |
| **Arañeae** |  |  |  |  |  |  |  |  |  |  |
| Amaurobioidea | Am1 |  |  | 8 |  |  |  | 1 | 1 |  |
| Anapidae | Apm1 |  |  |  |  |  |  |  |  | 2 |
| Anyphaenidae | MT1 | 1 | 1 | 1 |  |  |  | 6 | 2 |  |
|  | MT2 |  |  |  |  | 1 |  |  |  |  |
|  | MT3 |  |  | 1 |  |  |  |  |  | 1 |
|  | MT4 |  |  |  |  |  | 2 |  |  |  |
| Ageleniidae |  |  |  |  |  |  | 1 |  |  |  |
| Araneidae | AA1 | 1 |  |  |  |  | 1 |  |  |  |
| Cribelatae |  |  |  | 1 |  |  |  |  |  |  |
| Dictinidae | DM1 |  |  | 1 | 6 |  | 1 |  |  |  |
| Gnaphosidae | GM1 | 11 | 17 |  | 2 | 4 |  |  |  |  |
|  | GM2 |  |  |  |  |  |  |  |  | 11 |
| Linyphiidae | Lmspot | 1 |  |  |  |  | 2 | 2 | 8 | 1 |
|  | LM1 |  |  |  |  |  |  | 1 |  |  |
|  | LM2 |  |  |  |  | 1 | 1 |  | 1 |  |
|  | LM3 |  |  |  |  | 1 |  |  | 1 |  |
|  | LM4 |  |  |  |  |  | 1 |  |  |  |
|  | LM5 |  |  |  |  |  | 1 |  |  | 2 |
| Lycosidae | *Lycosa* spp. | 1 | 6 | 4 | 11 | 33 | 7 | 31 | 40 | 12 |
| Miturgidae | Rm1 |  |  |  |  |  |  |  | 1 |  |
| Pholcidae | Pm1 |  | 2 |  |  |  |  | 1 |  |  |
| Salticidae | *Saphrys* spp. |  |  |  |  | 1 |  |  |  |  |
| Scytodiidae | *Scytodes globula* |  |  |  |  | 1 |  |  |  |  |
| Theridiidae | TM1 |  |  |  |  | 1 |  |  |  |  |
| **Orthoptera** |  |  |  |  |  |  |  |  |  |  |
| Anostostomidae |  |  |  |  |  |  |  |  |  | 1 |
| Grillidae |  |  |  |  |  | 3 |  |  |  |  |
| **Coleoptera** |  |  |  |  |  |  |  |  |  |  |
| Anthicidae | *Vacusus holoxantus* |  |  | 6 |  |  |  |  |  |  |
|  | *Acanthinus* sp1 |  |  |  | 1 |  |  |  |  |  |
| Carabidae | *Criniventer rufus* |  |  | 3 |  |  |  | 58 |  |  |
|  | *Harpalinae* spp. |  |  |  |  |  |  |  |  | 1 |
|  | *Tetragonoderus viridis* |  |  |  |  |  |  |  | 9 |  |
|  | *Trirammatus striatula* |  | 3 |  |  | 3 | 10 | 5 | 2 | 2 |
|  | *Feroniola aerea* |  | 2 |  | 15 |  | 5 | 10 | 7 |  |
|  | *Nothocys* spp. | 28 | 65 | 106 | 19 |  | 32 | 24 |  | 3 |
|  | *Nothophena lonae* |  | 53 |  | 5 | 12 |  | 3 |  |  |
| Chrysomelidae | *Jansonius aenus* |  |  |  |  |  |  |  | 2 |  |
| Coccinellidae | *Eriopis connexa* |  |  |  | 1 |  |  |  |  |  |
| Corylophidae | Corylophidae sp1 |  |  | 2 |  | 81 |  |  |  |  |
| Cryptophagidae | *Cryptophagus* sp1 |  |  | 3 |  |  |  |  |  |  |
| Curculionidae | *Baridinae* |  |  |  |  |  |  |  | 1 |  |
|  | *Sphenophorus brevipennis* |  |  |  |  | 1 |  |  |  |  |
|  | Cryptorynchinae spp. |  |  |  |  |  | 1 |  |  |  |
|  | *Pentarthrum huttoni*? |  |  |  |  |  | 4 |  |  | 1 |
|  | *Scirpicola germinatus* |  |  |  |  |  |  | 1 |  |  |
|  | *Listronotus spp* |  |  |  |  | 1 |  | 1 | 1 |  |
|  | *Notiode*s sp1 |  |  |  |  |  |  |  | 1 |  |
| Elateridae | *Pseudoderomecus spp.* |  |  |  |  |  |  |  | 1 |  |
|  | *Llanquinue vittipennis* |  |  |  |  |  |  |  |  | 1 |
|  | *Deromecus curtis* |  |  |  |  |  |  |  |  | 1 |
| Heteroceridae | *Heterocerus* spp. |  |  |  |  | 5 |  | 1 |  |  |
| Histeridae | *Euspilotus* spp. |  |  |  | 1 |  |  |  |  |  |
| Languriidae | *Loboroschema bimaculata* |  |  |  |  |  |  | 1 |  | 1 |
|  | *Loberus* spp. |  |  |  |  | 1 |  |  | 2 | 2 |
| Lathridiidae | *Melanophthalma* spp. |  |  | 2 |  | 1 |  |  |  | 1 |
|  | *Cartodere* sp1 |  |  |  |  |  |  |  | 3 |  |
| Leiodidae | Leiodidae sp1 |  |  |  |  |  |  |  |  | 1 |
| Melyridae | *Arthrobracus nigrimaculatus* |  |  |  |  | 25 |  |  |  |  |
|  | *Hylodanacea derbesii* |  |  |  |  |  |  |  | 1 |  |
| Pselaphidae | *Achillia kuscheli* |  |  |  |  | 1 |  |  |  |  |
| Ptinidae | *Niptomezium* sp1 |  |  |  |  | 2 |  |  |  |  |
| Scaphidiidae | *Boacera chilensis* |  |  |  | 1 |  |  |  |  |  |
| Scarabaeidae | *Astralophorus frenchi* |  |  |  |  |  |  | 3 | 2 |  |
| Staphilinidae | *Carpelinus stricticollis* |  |  |  |  | 6 |  |  |  |  |
|  | Oxitelinae sp1 |  | 4 |  | 3 | 41 | 3 |  |  | 1 |
|  | Oxitelinae sp2 |  |  |  |  | 1 |  |  |  |  |
|  | Oxitelinae sp3 |  |  |  |  |  | 1 |  | 1 | 1 |
|  | Oxitelinae sp4 |  |  | 1 |  |  | 7 |  |  |  |
|  | Osorinae sp1 |  |  |  |  | 1 |  |  |  |  |
| Tenebrionidae | *Nycterinus quadricollis* | 1 |  |  |  |  |  |  |  |  |
|  | *Nycterinus abdominalis* |  |  |  |  |  |  |  | 4 |  |
|  | *Nycterinus* spp |  |  |  | 1 | 1 |  |  |  |  |
|  | *Thinobatis* sp1 |  |  |  |  |  |  |  | 18 |  |
|  | *Thinobatis rufus rufus* |  |  |  |  |  |  | 1 |  |  |
|  | *Thinodromus luteipes* |  |  | 1 |  |  | 1 |  |  |  |
| Trachypachidae | *Systolosoma breve* |  |  |  |  |  |  |  |  | 2 |
| **Hemiptera** |  |  |  |  |  |  |  |  |  |  |
| Miridae |  |  |  | 2 | 11 |  |  |  |  |  |
| Nabidae |  | 3 |  |  | 3 |  |  |  |  | 1 |
| Saldidae |  |  | 5 |  |  |  |  | 1 | 1 |  |
| **Homoptera** |  |  |  |  |  |  |  |  |  |  |
| Aphididae |  |  | 1 |  | 1 | 3 | 1 |  | 1 | 1 |
| Cicadellidae |  |  |  | 2 |  |  |  |  | 1 |  |
| Delphacidae |  | 1 | 2 |  | 7 | 1 |  |  |  |  |
| Pseudococcidae |  | 1 |  | 1 | 4 | 4 | 3 |  |  |  |
| **Hymenoptera** |  |  |  |  |  |  |  |  |  |  |
| Formicidae |  |  | 40 |  | 47 | 131 | 48 |  | 12 |  |
| **Lepidoptera** |  |  |  |  |  |  |  |  |  |  |
| Geometridae |  |  |  | 4 |  | 1 |  |  |  |  |
| Noctuidae |  |  | 1 |  | 2 |  |  |  |  |  |
| **Diptera** |  |  |  |  |  |  |  |  |  |  |
| Dolichopodidae |  |  |  |  |  | 1 |  |  | 1 | 1 |
| Stratiomyidae |  |  | 134 |  | 4 |  | 2 | 28 |  |  |
| Ceratopogonidae |  |  |  | 2 | 1 |  | 1 |  |  |  |
| Fanniidae |  |  |  |  |  |  |  | 3 |  |  |
| Muscidae |  |  |  |  | 1 |  |  |  |  |  |
| Tabanidae |  |  | 6 |  |  |  |  |  |  |  |
| Ephydridae |  |  |  |  |  |  |  | 2 |  |  |
