# Supplementary material for: Partitioning the effects of regional, spatial, and local variables on beta diversity of salt marsh arthropods in Chile
Source: Ecol Evol. 2019 Jan 30;9(5):2575–87. doi: 10.1002/ece3.4922 (PMC6405494; doi:10.1002/ece3.4922)
Supplement: Supplementary file 4 [file ECE3-9-2575-s004.docx]

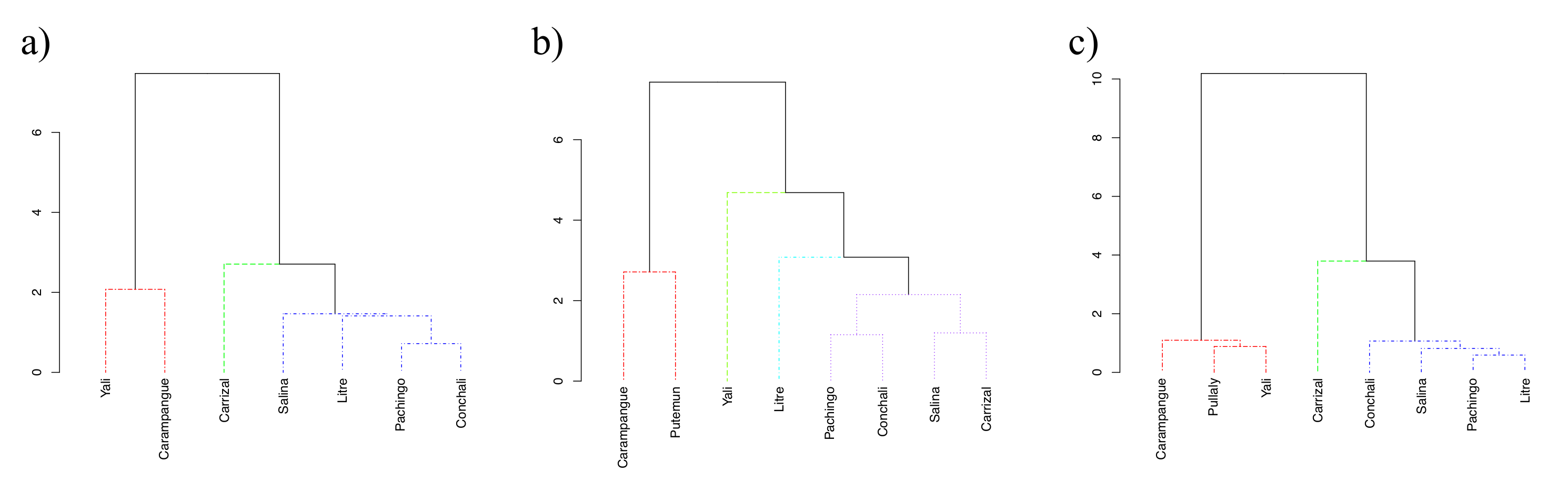


**Appendix 4**. Clusters analyses based on seasonal mean (a), autumn (b) and spring environmental data (c) showing the degree of similarity between marshes. Data include climate, edaphic and vegetation variables.
