# Supplementary material for: Partitioning the effects of regional, spatial, and local variables on beta diversity of salt marsh arthropods in Chile
Source: Ecol Evol. 2019 Jan 30;9(5):2575–87. doi: 10.1002/ece3.4922 (PMC6405494; doi:10.1002/ece3.4922)
Supplement: Supplementary file 5 [file ECE3-9-2575-s005.docx]

**Appendix 5**. Information for the global and individual fractions of all the dataset cosidered in the variation partitioning analyses (n =16) on beta diversity (Bsor) and its turnover component (Bsim). Global fractions: S = spatial; C = weather; E = edaphic; V = vegetation. Individual fractions: S/CVE; C/SVE; V/SCE ; E/SCV; S/CV; C/SV; V/SC; S/CE; C/SE and E/SC; S/E; E/S.

| Taxon |  | Source | adjR2 | Df model | Df Residual | F | P |
| --- | --- | --- | --- | --- | --- | --- | --- |
| Tot Arthropods | βsor | S | 0.121 | 0.044 | 0.203 | 3.074 | 0.001 |
|  |  | C | 0.120 | 0.044 | 0.203 | 3.057 | 0.001 |
|  |  | V | 0.104 | 0.055 | 0.192 | 1.879 | 0.001 |
|  |  | E | 0.113 | 0.042 | 0.205 | 2.914 | 0.006 |
|  |  | S/CVE | 0.054 | 0.022 | 0.126 | 1.776 | 0.042 |
|  |  | C/SVE | 0.025 | 0.017 | 0.126 | 1.366 | 0.173 |
|  |  | V/SCE | 0.052 | 0.035 | 0.126 | 1.411 | 0.085 |
|  |  | E/SCV | 0.008 | 0.014 | 0.126 | 1.118 | 0.369 |
|  | βsim | S | 0.053 | 3 | 12 | 1.283 | 0.004 |
|  |  | C | 0.060 | 1 | 14 | 1.971 | 0.001 |
|  |  | V | 0.029 | 1 | 14 | 1.454 | 0.01 |
|  |  | S/CV | -0.005 | 3 | 10 | 0.972 | 0.586 |
|  |  | C/SV | 0.014 | 1 | 10 | 1.177 | 0.252 |
|  |  | V/SC | 0.006 | 1 | 10 | 1.073 | 0.386 |
| Crustacea | βsor | S | 0.136 | 1 | 14 | 3.378 | 0.008 |
|  |  | C | 0.135 | 2 | 13 | 2.178 | 0.002 |
|  |  | E | 0.07 | 1 | 14 | 1.213 | 0.033 |
|  |  | S/CE | 0.028 | 1 | 12 | 2.2022 | 0.009 |
|  |  | C/SE | 0.053 | 1 | 11 | 1.4397 | 0.072 |
|  |  | E/SC | -0.0011 | 1 | 12 | 1.2212 | 0.279 |
|  | βsim | S | 0.072 | 1 | 14 | 2.1213 | 0.033 |
| Coleoptera | βsor | S | 0.182 | 2 | 12 | 2.558 | 0.002 |
|  |  | E | 0.118 | 1 | 13 | 2.881 | 0.004 |
|  |  | S/E | 0.11 | 2 | 11 | 1.9516 | 0.003 |
|  |  | E/S | 0.05 | 1 | 11 | 1.7656 | 0.064 |
|  | βsim | S | 0.086 | 1 | 13 | 2.346 | 0.002 |
|  |  | E | 0.082 | 1 | 13 | 2.2601 | 0.002 |
|  |  | S/E | 0.032 | 1 | 12 | 1.472 | 0.037 |
|  |  | E/S | 0.028 | 1 | 12 | 1.415 | 0.053 |
| Araneae | βsor | / | / | / | / | / | / |
|  | βsim | S | 0.073 | 1 | 13 | 2.103 | 0.002 |
